# eHealth interventions for psychiatry in Switzerland and Russia: a comparative study

**DOI:** 10.3389/fdgth.2023.1278176

**Published:** 2024-01-19

**Authors:** Olga Chivilgina, Bernice S. Elger, Ilya Fedotov, Fabrice Jotterand

**Affiliations:** ^1^ Institute of Biomedical Ethics, University of Basel, Basel, Switzerland; ^2^ Unit of Health Law & Humanitarian Medicine at the Institute for Legal Medicine, University of Geneva, Geneva, Switzerland; ^3^ Psychiatry Department, Ryazan State Medical University, Ryazan, Russia; ^4^ Center for Bioethics and Medical Humanities, Institute for Health and Equity, Medical College of Wisconsin, Milwaukee, WI, United States

**Keywords:** digital technologies, psychiatry, ethics, eHealth, technology adoption, mental health

## Abstract

**Introduction:**

During the past decade, the use of digital technology to promote mental health has increased dramatically. Additionally, the consequences of the COVID-19 pandemic, such as travel restrictions and the disruption of face-to-face interactions, have led to an increase in the use of digital technologies. A wide variety of technologies have been developed, including messaging chatbots, virtual reality technologies, direct-to-consumer apps, and even technologies that are fully integrated into clinical care tools.

**Methods:**

The following qualitative study is based on the opinions of mental health specialists in both countries regarding the use of digital health technologies in psychiatry in Switzerland and Russia in 2019–2020. We investigate the state of adoption of digital technologies in the field of mental health, the meaning of such technologies, and the crucial factors in the use of such technologies in psychiatry.

**Results:**

Health care professionals in both Russia and Switzerland are well aware of these technologies. However, the use of digital technology to promote mental health has taken different paths in these two health care settings.

## 1 Introduction

Electronic health (eHealth) technologies for mental health form a growing market encompassing various technological components, such as mobile health (mHealth) applications (1), wearables and sensors (2), consumer neurotechnologies (3), virtual reality systems (4), online platforms (5), care coordination systems (6), assisted living ecosystems (7), and telemedicine (8, 9). The significant growth of eHealth, accelerated by the COVID-19 pandemic, has been marked by increased funding, fast-track policy approvals, governmental priorities, public-private partnerships, and collaborative research efforts (10). In the psychiatric domain, the application of eHealth technology has exhibited substantial potential, for example, for online consultations, computer- or mobile phone-mediated cognitive-behavioral therapy (11), monitoring medication adherence (12), preventing disease exacerbation, symptom management (13), symptom reduction (14), interventions for the treatment of alcohol and substance abuse (15) and many other aspects (16).

### 1.1 Swiss and Russian health care systems

Russia and Switzerland create an interesting comparison because of their different histories and cultures. The two countries differ in terms of size, mentality, and the ways in which their health care systems are organized and financed.

1. The Swiss health care system is based on mixed private-social health insurance and financed through several sources. Health insurance companies are private enterprises that act as providers of mandatory health insurance and offer a variety of health insurance policies (17). The three categories of insurance status—usual, half-private, and private—result in differences in access to medical services. In particular, policyholders who choose a “traditional” package find that insurance companies reimburse only expenses related to health care provided by external providers. To address nonessential procedures, individuals in Switzerland can opt for supplementary insurance policies that operate within a voluntary private insurance system (18).

Some authors have criticized the Swiss health care system for its perceived unequal ability to provide both curative and preventive medicine to all individuals (19).

2. Russia, in contrast, features a highly centralized system focused on ensuring universal access to basic care. Under Russian law, health care, including psychiatric services, is universal, free and guaranteed as a constitutional right (20).

In light of the differences between these countries, it is unsurprising that the implementation of eHealth technologies varies.

The goal of our study was to understand how the use of eHealth in the field of psychiatry differs between Switzerland and Russia as well as the challenges associated with the implementation of such technologies in these countries. To address this issue, we explored (1) the current experiences of psychiatrists with eHealth use and their attitudes toward these technologies, (2) the eHealth technologies that are currently available for use in the field of psychiatry in both countries, (3) the structural and legal barriers to the widespread implementation of eHealth, and (4) possible solutions to ethical problems with the use of eHealth among psychiatric patients.

## 2 Materials and methods

A relevant group of senior-level psychiatrists and clinical psychologists working in Switzerland and Russia were recruited via email or phone. This group included senior-level psychiatrists or clinical psychologists identified either by reference to the websites of teaching hospitals in Switzerland or through recommendations from other mental health practitioners. A semistructured interview guide featuring open-ended questions was developed based on the literature (15, 21–25). The interviews were conducted in Switzerland from March 2019 to April 2020 and in Russia from January 2020 to June 2020. The Swiss interviews were conducted in English and German, and the Russian interviews were conducted in Russian. The interview guide featured open-ended questions and was sent to the participants via email prior to the interviews. All the interviews were conducted by the author, who was trained in the use of qualitative methods (OC). The interviews were continued until data saturation was reached, *i.e.*, until no new data emerged from subsequent interviews. The interviews each lasted for 35–55 min and were audio-recorded. Sixteen of the interviews with the participants from Switzerland were conducted in face-to-face meetings held at the interviewees' workplaces, and four were conducted via Skype. In Russia, six interviews were carried out in person and nine via Skype. The recorded interviews were then transcribed verbatim, and the Russian and German interviews were translated into English. The transcriptions and translations were spot-checked by other members of the research team. The anonymized transcripts were then imported into the qualitative data analysis management software MAXQDA. The usual standards for qualitative analysis in medicine were applied to the content analysis of interviews (26, 27). After the data familiarization stage, the data were categorized into codes that contained conceptually similar ideas or actions. Through an iterative process, the relationships between these codes were analyzed, and themes were formed by grouping related codes. We developed these categories inductively and followed a systematic set of steps for data analysis, including summarizing, explicating, and structuring. The figures were generated using the visual tools provided by MAXQDA.

## 3 Results

In Switzerland, emails were sent to 28 participants, 20 of whom agreed to participate in the study. In Russia, 30 individuals received invitations via email, and 15 agreed to participate. The participants' characteristics are described in the following table (Table 1, Participant characteristics).

**Table 1:** Participant characteristics.

	Switzerland	Russia
Work		
Hospital psychiatrists	15	15
- Chiefs holding a leadership position in a hospital department	7	4
- Senior psychiatrists	8	11
- Clinical psychologists	4	
Origin	12 from the German-speaking part of Switzerland (G)	4 Moscow, 3 St. Petersburg, 2 Ryazan, 1 Rostov, 1 Voronezh, 1 Sochi,1 Nizhny Novgorod, 1 Kazan, 1 Volgograd
	8 from the French-speaking part of Switzerland (F)	
Sex		
Male	15	12
Female	5	3
Age		
30–40	6	6
40–50	7	7
50–60	7	1
Older than 60	1	1

The use of digital technology was more frequent among participants from Switzerland than among their Russian counterparts (see Table 2). The most widespread type of eHealth technology used in Russia and Switzerland was mobile apps. In Switzerland, two participants reported using virtual reality technologies with their patients as part of a research project:

**Table 2:** The technologies mentioned by participants.

	Russia	Switzerland
Type of eHealth technologies used		
Mobile apps from the market	4	5
Self-developed mobile apps (or research in progress)	2	4
VR	-	1
Computer-based programs	-	2
AI	-	2
Wearable sensors	-	1
Telemedicine, telecommunication, including messengers	10	2
No experience using eHealth technologies	9 (60%)	7 (40%)
Total number of interviewees	15	20

P13, CH, G: “We are conducting a study with exposure-VR. Many patients hear a distressing voice in their head, but most of them are helpless against it. VR can add a second dimension to the therapy, e.g., that the patient is not only hearing the voice but can also visualize its origin and interact with that voice. The therapeutic idea is to give that voice a face and interact with that face via the therapist. So that the patient learns to control this voice, to change perspectives or adopt perspectives.”

The participant reported that they had used the “exposure” to virtual reality (VR) as part of cognitive behavioral treatment for unhealthy patterns, such as those exhibited by patients with anxiety or posttraumatic stress disorder. This practice aimed to confront such patients with a stressful situation in a simulated or controlled environment and to allow them to learn to control their behavior in a safe and confident manner. While these VR technologies possess the power to immerse the patient in a troubling virtual situation, they can also cause significant psychological trauma. At present, information regarding the short- and long-term physiological impacts of VR is scarce.

In both Switzerland and Russia, a significant number of the participants also discussed dilemmas related to the possibilities and pitfalls of the use of artificial intelligence (AI) programs in mental health care. In Switzerland, one participant was directly involved in the development of a clinical AI tool. This participant noted that these algorithms cannot provide a diagnosis; they can only contribute to clinical decision-making:

P14, CH, F: “Our aim is to create a proper AI that is specialized in mental health and that would actually be able to interact with people, and through this interaction, give proper data regarding the symptoms of the patient in a more accurate manner. So we are working right now on 2 types of AI: one does interesting research based on our online behavior, and the other is a proper AI speechbot.”

Data on the online behavior of patients can have a great impact on the diagnosis and treatment of mental diseases. However, information on mental health is often nuanced, and subjective and empathic skills are needed to interpret such information. Some participants were critical of the possibility that AI could understand the meaning of such information on its own:

P5, Ru: “It is clear that information on mental health has its own subtlety; it must be applied in the context of the patient's life, beliefs, and values as well as his environment, and the computer cannot interpret it.”

As technology continues to develop, decision support systems are expected to become even more refined and accurate with regard to making diagnoses and suggesting the correct type of treatment:

P13, Ru: “What should the sensitivity and specificity of the algorithm be in order to release it to the market? Now, there are no such algorithms that subtly understand 90% of the human psyche.”

Another participant was confident that AI could never act to promote patient welfare:

P15, CH, G: “Intelligence systems that never make any mistakes anymore, that are philanthropists, could never exist.”

The participants noted that the lack of transparency and interpretability of the processes by which AI results are generated renders the use of such technology in psychiatry particularly sensitive. The way an algorithm transforms input into output is often compared to a black box, meaning that it remains scientifically and practically opaque. There is no guarantee that AI will not produce harmful instructions or biased content. The interviewees were concerned that without understanding the justification for the decision made by an AI system, clinicians cannot trust its diagnoses or treatment recommendations:

P20, F: “If it's unclear how the AI understands and interprets information about the patient, how can I trust its decisions? Moreover, who of us is to blame when a mistake happens?”

Most of our study participants agreed that at present, the problem of responsibility for clinical decision-making based on AI remains unsolved. Thus, the implementation of technologies using AI remains very limited due to the corresponding technical, ethical, and legal issues:

P2, CH, G: “And also the question, ultimately, of responsibility and obligations on the part of medical professionals, for example, to intervene if the person is self-endangered as part of the mental illness and moves alone in a digital space.”

While discussing digital technologies, most of the Russian participants referred to telemedicine. In the context of a large country such as Russia, telemedicine can facilitate access to mental health care, especially in remote areas. Several participants commented on the successful use of telemedicine in their practice. They reported using teleconferencing both for remote consultations with patients and for communication among health care professionals in several hospitals:

P3, Ru: “All regional centers in Russia are divided between two medical research centers. Each center has its own telehealth department, so if there is a difficult case somewhere in a region, they can get in touch with the center and consult a patient there using a special protected system with a special type of communication to protect all transmitted data. So far, the reviews are positive: it's quick, affordable, and convenient.”

P2, Ru: “Recently, I’ve had a patient whose entire information from checkups, EHR [electronic health records], and data were sent to [the main research center] with the consent of the patient. We received very qualified recommendations that not only helped me choose a certain medicine but also provided me with extra arguments in order to prove the necessity of prescribing that expensive medicine.”

However, according to some participants, the use of telemedicine remains limited as a result of several factors, such as legal barriers. Doctors from another hospital are either unaware of the opportunity or do not use it due to concerns about data privacy:

P8, Ru: “For some of our patients, it will take about 4 h to visit the regional center. They can receive psychotherapeutic care only there. Today, many regional centers don’t exist anymore, so patients with panic disorder or anxiety disorder cannot get any cognitive-behavioral therapy or any other help. It would also be useful if it were possible to organize consultations online using telemedicine from time to time and to treat patients together with local doctors. Still, there are conditions for it; we just do not use them. Such sessions are not in place because there is no basic understanding of our opportunities.”

P10, Ru: “I haven't heard about such technology being officially used in public health care. In general, it is prohibited to carry out consultations by phone or using social media to avoid disclosure of confidential data.”

Several of the participants from Switzerland highlighted the importance of telemedicine for providing better access to psychiatric health care when facing an unequal distribution of health care services:

P 19, CH: “Compared with other countries, Switzerland is a country with a high concentration of psychologists and psychiatrists. Still, not everywhere do people have equal access to their services That is mainly due to the incorrect resource allocation: the highest density of health care specialists is concentrated in the big cities. So in [name of a canton], there are 10 times more psychiatrists per capita than in [name of another canton]. Furthermore, [region of a canton] has only 2 practicing psychiatrists for the whole population. The implementation of telemedicine could improve the psychiatric care landscape in Switzerland.”

Some doctors mentioned that they occasionally conducted psychotherapeutic consultations online, often due to the distance between them and the patient. However, they noted that such practices can compromise data security if they are organized without ensuring the safety of user data pertaining to mental health. Some online tools do not offer sufficient privacy protection regarding health data, which means that the manufacturer can share or sell user data for marketing or other purposes. This issue is exacerbated by the lengthy legal text that makes up a privacy policy, making it hard for the user to understand or foresee possible consequences:

P1, Ru: “If online therapy is performed via Skype, WhatsApp or any other messenger or whatever means, there may be violations of doctor-patient confidentiality. When agreeing to such consultation, patients should be very much aware of this fact.”

**Figure 1:**
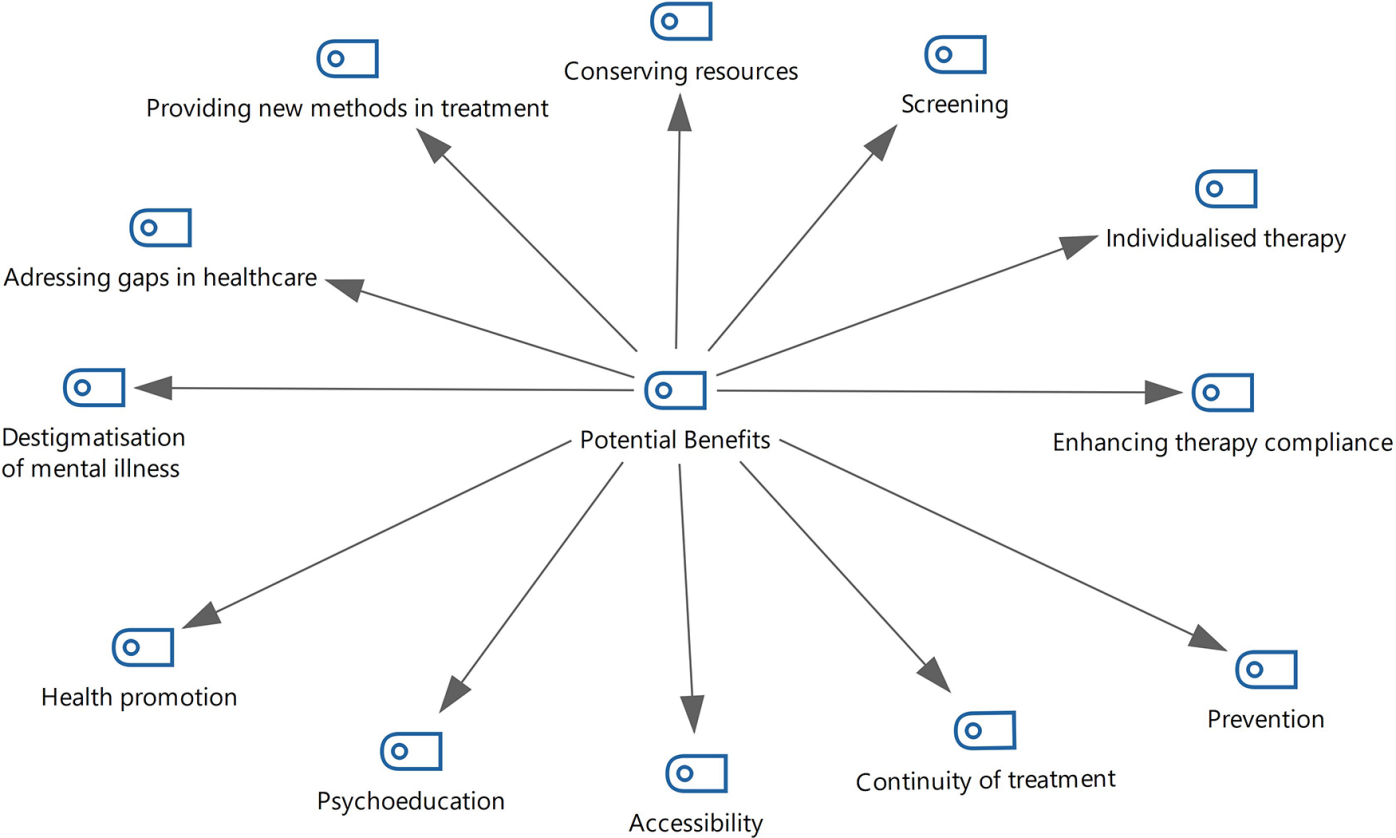
Benefits of eHealth.

Overall, all participants mentioned certain positive aspects of eHealth technologies, as they may be used to add value to patients' care (Figure 1, Benefits of eHealth).

For example, the use of digital technologies can help patients overcome the stigma associated with visiting a psychiatric hospital. In turn, this lack of stigma may improve compliance with therapy and mental health awareness:

P7, CH, F: “For me, the technology seems to be helpful for the treatment, and I see more positive effects. Patients and doctors gain better knowledge of the illness, patients have more and better compliance, and maybe for them, it could also be useful to see another patient speak about his condition. Also to have the hope to come out and cope with their mental health issues.”

The interviewees reported that eHealth can complement face-to-face sessions, support the continuity of treatment, and bridge the gap between health care specialists and patients by providing extra support when face-to-face therapy is unavailable. Thus, patients with long-lasting mental disorders may benefit from the use of digital interventions to complement their regular therapy:

P2, CH, G: “With technological support between face-to-face contacts, the therapeutic process is likely to be more continuous.”

In the case of therapeutic games or virtual realities, eHealth provides new therapeutic tools that may contribute to improving the effectiveness of therapy:

P13, CH, G: “The technology can bring a second dimension to the therapy. Using VR, patients who hear a voice can also visualize the origin of this voice and work on it.”

**Figure 2:**
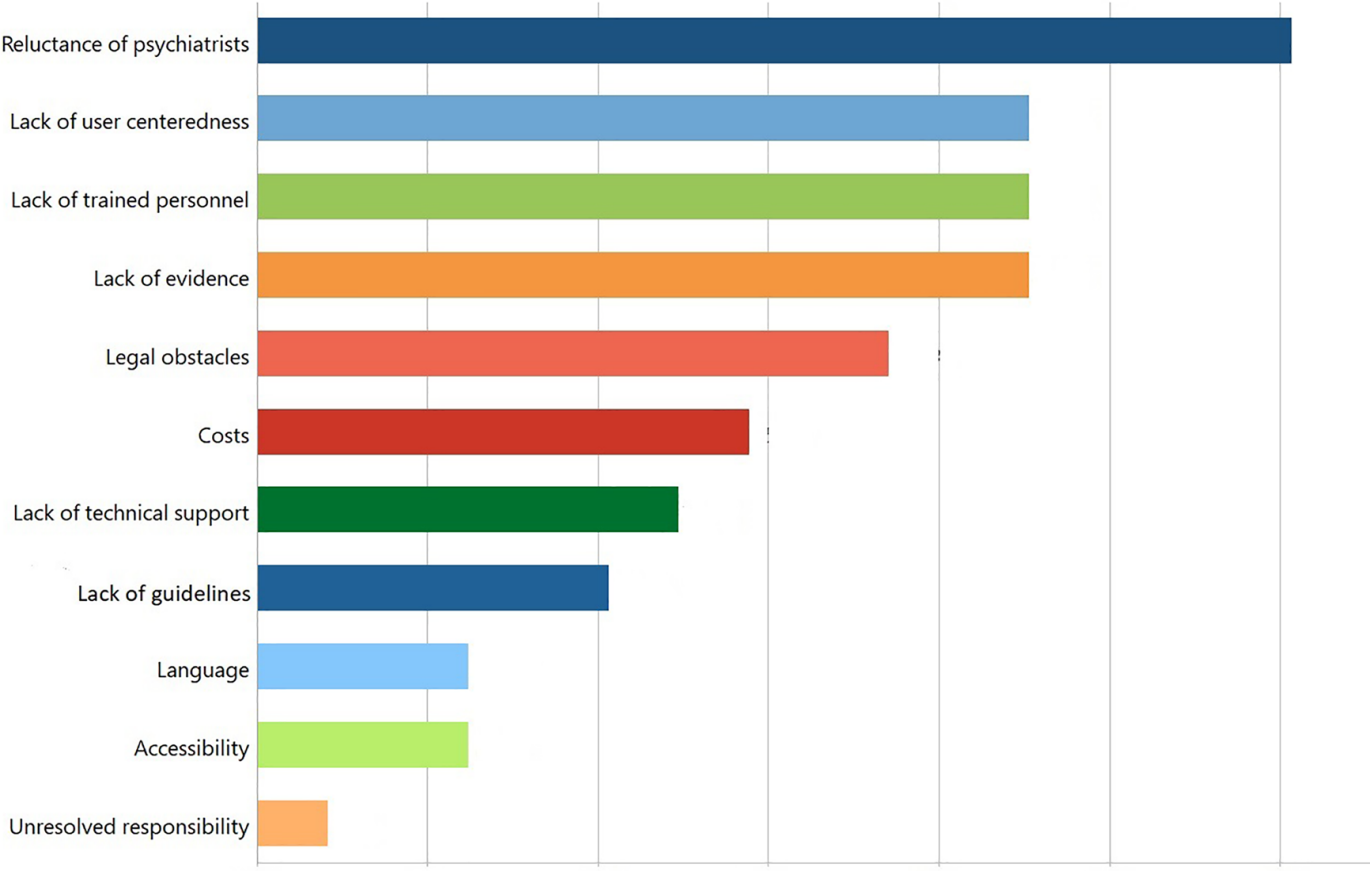
Obstacles in implementing eHealth.

The participants also mentioned various obstacles that impeded the implementation of eHealth in clinical practice (Figure 2, Obstacles in implementing eHealth).

Although many apps have been developed to promote mental health, their practical use remained scarce in both countries due to many obstacles, as exemplified by the following statement:

P12, CH, F: “If you search in the literature, there are plenty of technologies that work. When you see real life—here or in other places—most of them are not used because it's difficult to integrate them into the clinical setting. Sometimes, it's, yeah, it's difficult to know what was the reason.”

Possible reasons for the lack of applications in practical use were accessibility and usability issues, legal obstacles, costs, lack of research support, lack of evidence and clinical recommendations, skepticism, and technical issues. These issues are addressed in detail in the next subsection.

### 3.1 Accessibility and usability

Some participants highlighted the importance of a user-centered design for the successful adoption of technology by patients. The interface of eHealth technologies should be intuitive, engaging, and straightforward, as mentioned by one participant:

P15, CH, G: “For someone who doesn’t have a clue about psychiatry and simply downloads the app as a patient, some of the suggested exercises could be difficult.”

The participants were aware that the language used by an app to communicate with patients is a crucial aspect of a user-friendly design. The language should be nondiscriminatory; e.g., it should avoid transferring social normality bias or discriminating against people with mental health conditions.

According to the Russian participants, only a minority of the available technologies were available for Russian-speaking users. In the context of non-English-speaking countries, it is important to provide a service in the local language; otherwise, the uptake rate of the technology by the population will be low, and its usefulness will be hampered:

P2, Ru: “Unfortunately, in Russian segments of the internet, there are very few applications or self-help programs in Russian. So I have to encourage my patients to use simple versions in English. Usually, in such applications, they keep their diaries using smileys, and that is clear.”

The development of commercial apps occurs predominantly in Western countries; consequently, such apps are mostly oriented toward Western customers. The poor understanding of non-native-language users restricts their understanding of the instructions, feedback, terms of use and privacy rules of these apps. In some cases, psychiatrists lack suitable alternatives and “make do” with what is available, leading to a possible subpar outcome compared with applications in their patients' native language.

In the opinion of some interviewees, older patients may be less likely to benefit from digital technologies for their treatment:

P12, CH, F: “Based on our observations, there were very few patients who didn’t benefit from the intervention. The only characteristic that these patients had [in common] was age.”

The participants expressed concern about the possibility that with more widespread use of technology, the gap in quality and access to care between digitally literate and digitally illiterate patients would increase. The fact that older people tend to use technologies less frequently should not exclude them as potential users of eHealth technologies. Multiple studies have shown that elderly people can use technologies well if they are provided with initial instructions and good technical support (28). Additionally, a user-centered design and intuitive interface are important for promoting adherence to technology:

P7, CH, F: “It would be unethical to develop something that is accessible only by a small proportion of patients. It should also be very clear that the information is easy to understand and not too boring so that people can actually use the technology.”

### 3.2 Legal obstacles

Many of the Russian participants noted the problems facing existing legislation on telemedicine, which naturally restricts the implementation of telemedicine by discouraging both doctors and patients from using digital tools to initiate and continue treatment (Legislation on Telemedicine, (29). For instance, diagnosing a patient remotely and prescribing medications during the first appointment are prohibited. Medications may be prescribed only on the basis of a face-to-face meeting between the physician and the patient:

P9, Ru: “One opportunity I’d be happy to have is to be able to prescribe a medicine without my patients necessarily being present. Unfortunately, patients still have to come personally to receive a prescription, or we apply to a delivery service. I wish we could do it online the way it has been made possible in Moscow.”

Furthermore, some of the Russian participants indicated the importance of detailed consent. In their opinion, the legal basis for electronic consent in eHealth is insufficient. The laws and regulations for the protection of medical data and evaluating the decision-making capacity of patients can be deceptive:

P5, Ru: “If the person did not sign an informed consent online and his interaction is not a medical appointment, he just receives a consultation; this is a regular service sector, regulated by consumer protection law. But this is not accountable to 323 of the federal law on health protection.”

P1, Ru: “This is a loophole in the legislation because an app is not a human and cannot evaluate the age and decision-making capacity of the user who enters his or her data.”

Another participant was concerned that medical data can be legally accessed by third parties, especially by law enforcement, which can lead to stigmatization of and discrimination against individuals with mental health conditions:

P6, Ru: “A delicate issue is that an app is often attached to a telephone number. Access to the numbers is available to the law enforcement bodies. Once you’ve downloaded an app, you’ll be considered a person with some problems, already belonging to some risk group, and these apps could become an instrument of surveillance and label attaching.”

The opinions of participants about the legal regulations in Switzerland varied. The area of eHealth is regulated at different levels: the handling of personal data is regulated by the Data Protection Law (DSG), while the implementation and clinical safety of such technologies are under the control of Swissmedic. According to some participants, the DSG does not provide adequate protection for mental health data:

P8, CH, F: “I think digital technologies in psychiatry are not sufficiently regulated. There are regulations on data protection in the federal law. We have a legal basis, but I think it's not enough for mental health. People with mental health issues are a vulnerable population, and they need more protection than other patients.”

Given the sensitivity of mental health data, such data also represent a target for cybercriminals. In March 2022, the private medical data of many citizens were released on the darknet following a cyberattack on private practices in the French-speaking region of Switzerland. Official statistics concerning data security breaches or data leaks in Russia are not reported to the public.

The participants from both countries expressed concern about the privacy of information registered in digital technologies as well as the security of such information and the purposes for which it would be used.

Some interviewees were concerned that the database created by eHealth technologies for mental health may eventually be at risk of misuse. While passive monitoring of these data may allow for preventive interventions and thus an overall better outcome for the patient, it may also lead to social profiling. In particular, the conflation of mental health with criminal behavior may lead to a discriminative categorization of people that violates the principle of justice, undermines human rights, and leads to unfair outcomes.

Passive psychological monitoring can be used to detect changes in the mental states of patients. The participants from both countries discussed the implementation of such technologies. The early identification of mental disorders or the exacerbation of diseases based on the identification of behavioral patterns would benefit patients. These preventive interventions before the onset of illness can potentially prevent a more pernicious course of the disease and promote patients' quality of life. However, evidence regarding the clinical utility of such interventions requires further investigation. Prediction algorithms assisted by machine learning require legal regulation because such algorithms can also be misused. In particular, social profiling and the conflation of mental health with a crime may lead to the discriminative categorization of people, which violates the principle of justice, undermines human rights, and leads to unfair outcomes.

P14, CH, F: “The way we behave on the internet, on our mobile phones, the way we deal with technology and use it in our daily life, might have an impact on our mental status. The most interesting part is that this behavior is not conscious. If we could identify those unconscious patterns of use and link them to mental status, then we would be able to detect someone who is depressed before himself. And what that would allow—it would allow the technology to connect them to specific therapists who will be able to intervene to prevent depression.”

P5, Ru: “Neuroprediction techniques are of major interest, and there is still a great need to understand how they can be implemented, for example, in risk assessment in the field of forensic psychiatry.”

Another interviewee commented on the complexity of the current regulations stipulated by Swissmedic, the Swiss surveillance authority for medicines and medical devices: while the registration process for technology is difficult or even prohibitive for local developers, an abundance of non-evidence-based consumer eHealth apps are available online with little oversight:

P15, CH, G: “If we want to develop medical apps, we have to provide plenty of data on effectiveness and safety to Swissmedic. And if Swissmedic makes life so difficult for us, it could put a certain amount of regulation on all the other health apps in the app store, but that is extremely resource-intensive. But if they did random sampling and simply blocked certain apps, you couldn’t upload every nonsense online. Then, maybe the quality of the available digital interventions would just improve over time.”

### 3.3 Costs and accessibility of technology

In both Russia and Switzerland, the participants mentioned that eHealth is a cost-saving option for therapy. There is increasing evidence that many eHealth technologies, especially apps, are beneficial in terms of providing care to broader groups of patients:

P6, Ru: “As we are living in the epoch of compromises, this is a kind of a middle ground. Sometimes the cost of traveling to my office is equivalent to the cost of a consultation itself. Why should a patient pay twice?”

P8, CH, F: “eHealth can decrease the costs of health care. Even when implemented as an adjunct within a psychotherapeutic setting, an online intervention gives the therapist more time to address more complex and individualized issues with the patient. At the same time, the patient can learn and practice some skills online.”

However, the welfare of patients must be prioritized over economic benefits. The implementation of digital tools and embedding technology as an element of health care in clinical practice requires a complex ecosystem with the patient at the center. eHealth should not be viewed as simply another way of saving costs or as a substitution for face-to-face therapy.

The participants from both countries noted the high prices of some apps. High costs can be a barrier to entry for unemployed patients or patients facing financial difficulties. A study on the adoption of mental health apps showed that app prices are significantly negatively correlated with installation rates and app ratings (30). This indicates that for the successful integration of technologies into health care, the development of reimbursement mechanisms is needed:

P12, CH, F: “Most of our patients don't have a lot of money or work. They receive very little money from social services and have very restricted insurance, and they cannot afford to buy a smartphone or a paid subscription for an app.”

The approaches that countries take, or can take, toward app reimbursement vary in accordance with their respective modes of health care financing.

In Russia, the health care system is financed primarily by the state. A separate category, so-called high-tech care, is supported by the government. However, whether eHealth may be included in this category remains unclear:

P3, Ru: “Both options, free and paid applications, should be available. The paid option is for those who turn to private clinics. And the applications which were recognized by the parties responsible as needing this ‘high-tech care’ should be provided free of charge.”

In Switzerland, private stakeholders such as insurance companies are interested in providing new ways of supporting the mental health of customers through the use of eHealth technologies. Embedding eHealth in prevention programs may optimize and reduce total spending on mental health care:

P15, CH, G: “Insurance companies want to save money by focusing on prevention. These are the important stakeholders in Switzerland that are interested in supporting technology development for the benefit of patients.”

This might, however, come with a significant risk: some of the participants raised concerns that the involvement of insurance companies may lead to conflicts of interest between the welfare of the patient and economic benefits. For example, insurance companies may be interested in saving costs and substituting the sole use of mobile health applications for face-to-face treatment, despite the apparent lack of maturity and privacy concerns mentioned previously:

P9, CH, F: “It's important that we keep the support from the state as much as possible, that it doesn't get overtaken by private groups with their own economic interests, such as health insurance companies. It's important that we keep in mind what is most useful for each patient, not only how we can save as much money as possible.”

### 3.4 Lack of research support

Many interviewees from Switzerland felt that there was a lack of support for clinical research and for practical implementations of eHealth technologies in clinics:

P15, CH, G: “Unfortunately, many of the technologies that are investigated fail to become established in the market. Whereas those that manage to enter the market are often not evidence-based. And now there are also efforts here in Switzerland to improve that. But that's relatively at the beginning.”

One interviewee noted that the high costs of the development of apps and clinical studies in Switzerland limit local technology development:

P14, CH, G: “App development and clinical studies in Switzerland have extremely high costs. So I might pay some programmer in another country and then just put an app online. And there is the need for more support to facilitate the quality that the specialists here can deliver.”

### 3.5 Lack of evidence and clinical recommendations

The lack of evidence-based technologies available on the market has been mentioned by many authors (15, 31). Ambitious marketing promises as well as fuzzy boundaries between clinically proven and non-evidence-based “wellness” apps can confuse both patients and clinicians:

P13, Ru. “There are many programs out there. There is also evidence that some of them help. But there are more apps without any evidence.”

Along with a lack of empirical evidence in this context, there is also a lack of national recommendations or clinical guidelines regarding eHealth technologies:

P15, CH, G: “Choosing a proper app for treatment is difficult. It would be useful to have guidelines with certain criteria based on the scientific publications that could help us estimate whether the technology will have a good effect.”

### 3.6 Skepticism of health care personnel

The implementation of eHealth has been further restricted by the hesitation, lack of acceptance, and even unwillingness of health care professionals.

The necessity of training hospital staff in the implementation of eHealth with patients was identified as a key factor for acceptance by many participants; a lack of knowledge fosters reservations and nonacceptance. This lack of knowledge regarding such technology is explained partly by the lack of clinical guidelines and national recommendations mentioned above:

P14, Ru: “I think one other problem is that you have to train doctors, nurses, and other therapists, and that is also a big problem to implement this in the clinic.”

The second human factor limiting the use of eHealth technologies in both countries was practitioners' lack of openness to the new technologies and unwillingness to prescribe them:

P1, CH, G: “I am scared of using technology instead of establishing a relationship. Technology instead of conversation, technology instead of thinking together and seeing what happens in me when you say that. Something that belongs between people is being externalized, digitalized.”

A prominent argument voiced by critics against the use of such technology is the “special” therapeutic relationship between a health care specialist and a patient in the field of psychiatry and the importance of face-to-face contact in therapy:

P5, CH, G: “I find the most difficult ethical issue to be a certain diffusion of roles in the therapeutic relationship. Normally, the therapeutic relationship is quite clear; there is a professional and a patient. And ideally, they should speak with eye contact, respect and with each other—that is the ideal situation. But if I now have an app that is interposed like a 3rd party in therapy, that changes these roles.”

Considering the dynamic development of medicine, each clinician should reflect on how the use of such technology may impact the therapeutic relationship based on its benefits and limitations. For the benefit of patients, mental health providers must be informed of all existing options for improving the mental well-being of their patients. This includes evidence of the efficacy and possible adverse outcomes of the use of such technology as well as potential issues with data security and the ethical use of the technology.

### 3.7 Technical issues

Several of the Russian participants identified the lack of technology to support data encryption and safe data transfer as important factors in the poor uptake of eHealth in psychiatry:

P13, Ru: “Unfortunately, insufficient technical equipment is a huge problem. We do not have so many servers, and we do not have so many secure encryption channels or data transmission systems.”

Swiss psychiatrists spoke about the challenge of protecting data security when confronted with potential future opportunities to interconnect mobile apps and fuse data from multiple sensors. An increase in connectivity also carries an increased risk of data confidentiality breaches:

P11, CH, G: “Another step would be to connect the digital interventions with the hospital information systems. Data security is a big issue because of the particular sensitivity of this kind of personal data.”

A lack of the technological equipment and knowledge necessary to ensure data security can serve as a barrier to entry for the technology. Moreover, the implementation of such technology without ensuring the security of sensitive data is unacceptable.

### 3.8 Maximizing the potential of eHealth

**Figure 3:**
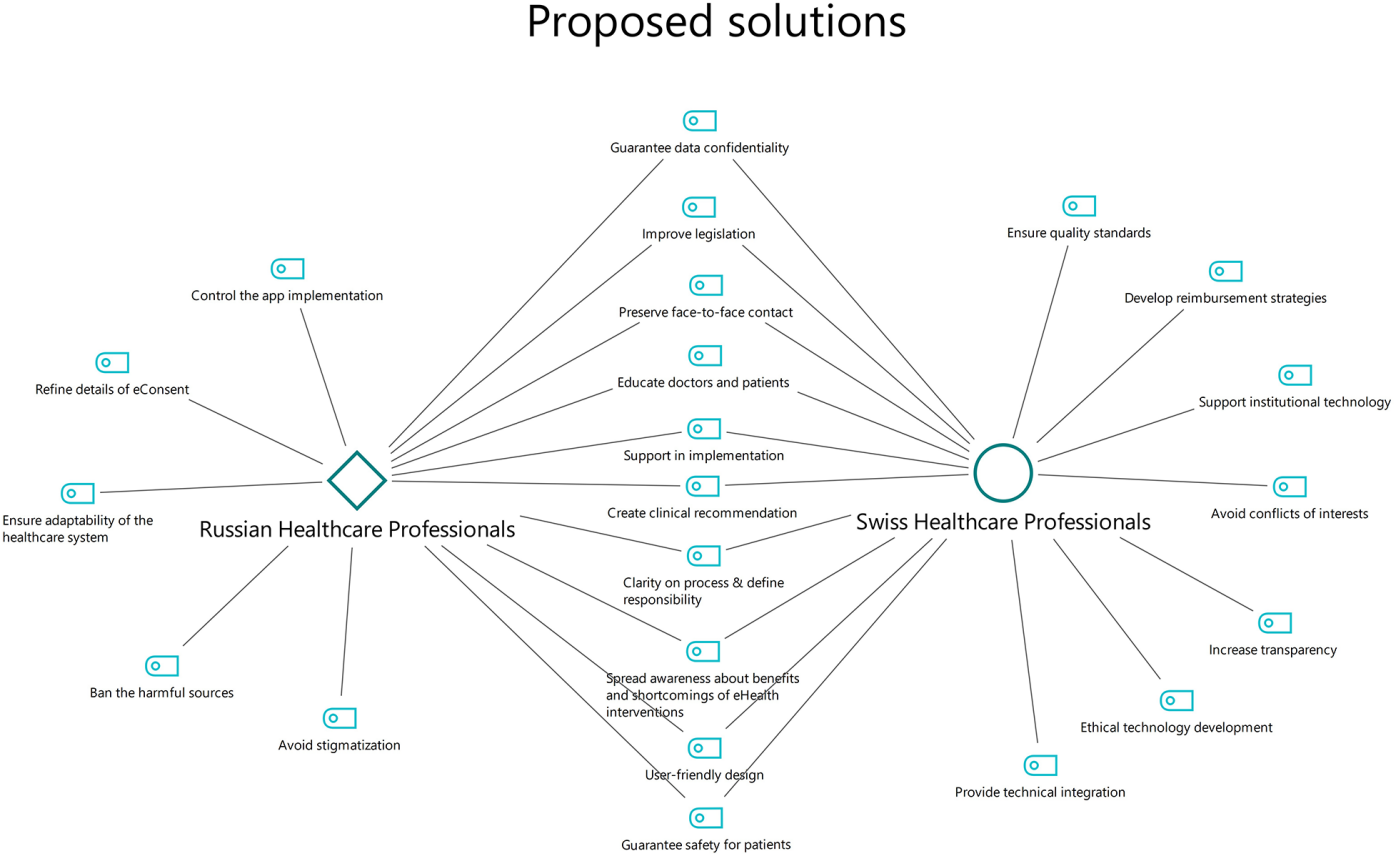
Proposed solutions.

To overcome these obstacles, the interviewees demanded significant changes. (Figure 3, Proposed solutions): Primarily, clinical recommendations and detailed governance of eHealth for psychiatry must be provided. They suggested creating practical solutions to address the problem of responsibility for and accountability of technology for safer and more effective implementation of technology in practice.

The participants emphasized the importance of continuing face-to-face contact between patients and health care providers while using digital technologies for mental health. Training and education for both health care specialists and patients who implement the technology should also be provided.

Technological innovation to promote mental health in both countries seemed to be developer-driven; accordingly, many of the participants highlighted certain limitations regarding the design and usability of eHealth technologies. Therefore, collaboration among developers, health care professionals and patients is necessary for the effective application of these technologies.

Many of the interviewees from both countries mentioned a lack of support from various levels of government, ranging from a deficiency in research financing to the scarcity of technological support for ensuring data privacy to problems with reimbursement mechanisms for technology costs. The legal basis for guaranteeing the safety of behavioral and health-related information must be improved, and the technical requirements of such security must be met. Health care providers need clear clinical guidelines for technology and corresponding reimbursement mechanisms to use such technologies with their patients.

The Swiss participants suggested more support for institutional researchers and sustainable technology development aimed at beneficence as well as reimbursement strategies for implementing technology. Transparency and avoiding or resolving conflicting interests were also identified as important factors in improving the implementation of eHealth interventions.

The Russian interviewees emphasize that the national health care system must be flexible and ready for digital transformation. The participants demanded that improvements be made to eHealth legislation, including specifying requirements for electronic consent and liberating telemedicine law.

## 4 Discussion

In the era of the digital transformation of health care, eHealth technologies are increasingly being used in the field of psychiatry. Our participants reported their use of diverse digital technologies and discussed the potential of such technologies in health care. eHealth can enhance therapy by increasing patients' autonomy and involvement in treatment. The digital interventions mentioned included monitoring apps, diaries, and tracking devices, which allowed patients and health care specialists to obtain more accurate knowledge of a particular patient's psychological patterns. Some digital technologies, such as virtual reality technologies and avatars, were able to produce and simulate real-life scenarios. The participants discussed the use of artificial intelligence technologies to support clinicians in diagnostic and clinical decision-making processes. This corresponds to the growing body of literature on mental health research using eHealth technologies in Switzerland and Russia (32, 33). A fairly large proportion of the respondents (40% in Switzerland and 60% in Russia), however, had not used eHealth. While many people use health apps, a substantial portion of mental health specialists do not use digital technologies with their patients. Common reasons for not using such technologies in both countries included the lack of evidence; the lack of legal and clinical recommendations; the incompatibility of the apps with clinical needs due to design, accessibility and usability issues; the high costs of research and technical development; the skepticism of health care personnel; and the lack of support for technology implementation. In Russia, additional challenges were the language barrier and a lack of technological resources to support the integration of technology.

The landscape of eHealth technologies differs between these two countries. We can explain this difference in light of several factors. In the setting of a large country such as Russia, there is a demand for low-cost applications that address the most common psychological problems on the widest scale possible. We observed advanced access to and use of mobile apps, ongoing research projects on AI and virtual technology in Switzerland and a broader use of telemedicine in Russia. Analytical articles have found that telemedicine consultations are the most convenient and permissible within the juridical borders of Russia (34). The results of telemedicine consultations have indicated that approximately half of patients receive a more accurate diagnosis, and more than 80% receive more effective treatment (35). Therefore, countries such as Russia are promoting the growth of low-budget technologies. In Switzerland, there is a wide variety of developed applications, although the concept of the integration of such technologies into clinical practice is lacking.

The current eHealth market has provided a wide range of digital solutions aimed at supporting the treatment of diverse psychiatric conditions. Numerous studies have demonstrated the efficacy and favorable usability of these interventions (15, 36). The costs associated with these technologies vary; some interventions, such as mobile applications or online treatments, when combined with traditional therapy, may be more cost-effective at preventing relapse than treatment through acute psychiatric hospitalization. The use of eHealth holds the promise of improving health outcomes for individuals living with chronic diseases by providing enhanced symptom control (37). Therefore, these eHealth technologies have the potential to be applicable in both settings.

Ethical issues in the use of medical applications played an important role for psychiatrists from both countries. Overall, many of the ethical and practical issues, such as concerns about data privacy, confidentiality, and access to technology, were perceived similarly in both countries. It is meaningful for health care specialists in both countries for technologies to be effective and safe for patients. The psychiatrists attached great importance to maintaining a trustful therapeutic relationship with the patient alongside the use of technologies as well as the concept of responsibility in patient treatment and the implications of technology for patient autonomy. Most of the interviewees spoke about discipline-related issues that hinder the implementation of technologies, where personal contact should be preferred.

According to the participants, the primary actions needed in both countries are refining laws and recognizing the costs associated with technology. In the national health care system, there are high expectations for government initiatives to provide countrywide recommendations and strategies for reimbursement and even to impose bans on harmful sources. Some Russian participants suggested that the legal definition of eConsent, particularly concerning the online processing of mental health data, should be more precisely specified.

## 5 Conclusion

Many factors determine the ways in which eHealth technologies are currently implemented in these two countries and the pathways taken by each country toward incorporating such technologies into mental health care. Certain common issues that are present in both contexts, such as the lack of user centrality in eHealth applications and the lack of inclusiveness in the use of technology to support mental health, have made such technologies difficult to implement.

Clinicians, researchers, and technology developers must take ethical considerations into account to reduce the risks and harm of such technology. To ensure the safety of patients, it is advisable that these technologies be used in specialized medical institutions under the guidance of trained health care professionals. The disruptiveness of eHealth was viewed with a great deal of personal skepticism by some health care experts. In fact, the place and role of such technology in the chain of health care professionals are undefined. Some technologies can be potentially harmful if used without the supervision of the therapist, for example, virtual reality or self-diagnostic tools. Information on the benefits and shortcomings of technology can be useful for shared decision making, selecting the most appropriate technology for treatment, and knowing how to mitigate potential risks and avoid harm.

To ensure nonmaleficence regarding patients, evidence-based technology is necessary, which requires additional support for national research and responsible software development. Responsible software development refers to the creation of technologies that promote the welfare of patients and aim to avoid causing potential harm. The design of technology to support mental health care should include user-centered and nondiscriminatory interfaces.

## 6 Limitations and directions for future research

One limitation of this study is that the participants were connected to university hospitals, so they may have had more favorable attitudes toward technology than other practitioners; however, they did report several concerns about the acceptability of eHealth technologies. Second, the uneven distribution of participants in terms of sex and age may have influenced the findings. Third, the study was conducted in Russia and Switzerland in 2019–2020. Prior to this time, not all of the participants had experience working with eHealth technologies. Even though we achieved data saturation, the modest sample size remains a noteworthy limitation, potentially constraining the generalizability of the findings.

Our study shows that specialists from both countries agreed that eHealth technologies should be adapted to different categories of patients. It is important to collect diversified data regarding the outcomes of the use of such technologies by patients to avoid the gray zone of off-label use. Collecting these data is essential for providing clinical recommendations to health care professionals. Services with differentiated functionality and composition are needed for different age categories and diagnoses. People with mental disorders have special needs, and their satisfaction with the digitalization of mental health services is an important ethical aspect in the relationship between specialists and patients. We advocate for the development and evaluation of eHealth technology in light of the variable conditions of health care settings and by reference to a wide range of participants since the population of users of this technology can be diverse. The elderly population as well as patients with serious mental conditions are often not included in studies because of the incentives of the developers of eHealth technologies to bring the technology to market quickly rather than to conduct multiple studies with different groups of patients. Future studies should also be conducted with patients who use eHealth technologies to improve our understanding of the value added to patient care by such technologies.

## Data Availability

The raw data supporting the conclusions of this article will be made available by the authors, without undue reservation.
