# Supplementary material for: eHealth interventions for psychiatry in Switzerland and Russia: a comparative study
Source: Front Digit Health. 2024 Jan 19;5:1278176. doi: 10.3389/fdgth.2023.1278176 (PMC10834775; doi:10.3389/fdgth.2023.1278176)
Supplement: Supplementary file 1 [file Datasheet1.docx]

Interview Guide for the study participants

Research topic: what are the attitudes of psychiatrists and healthcare experts towards the implementation of digital eHealth technologies?

1. **Introduction**
2. I would like to begin by understanding your work experience in the field of psychiatry.

The information gathered about the participants:

1. Name
2. Age
3. Gender
4. Institution (hospital, private practice)
5. Present Position/post
6. Formal training in which domain: Psychiatry (specialization), clinical psychology

**B. Using eHealth with patients**

1. What type of eHealth (electronic healthcare) solutions such as smartphone applications or other electronic devices do you use in your medical practice?
   1. *Are there special technical approaches that you apply in therapy with your patients? (examples)*
2. With what kind of your patients do you use eHealth?

*Prompting questions:*

*A. How many of your patients have you recommended to use this type of apps or internet based symptom treatment offers for psychiatric patients?*

*B. How many in your opinion are using such eHealth technology without recommendation?*

*C. How many of your patients would you say are interested in adopting eHealth (electronic healthcare) solutions in their care?*

*D. What are the socio- demographics of your patient population? Which age and social status are they?*

10. In your experience: did you observe any positive/or negative outcomes of the use of these eHealth technologies for people with psychiatric diseases?

**C. Psychiatrists’ experience working with eHealth?**

11.Are your patients provided with trainings before using any eHealth technologies?

- 1. *(If yes: in what consists the training exactly? (risks, effects))*

12.To which patients with schizophrenia would you recommend mobile apps/websites and to which ones you wouldn’t?

- 1. *(What are the reasons?)*

In which areas of your work do you see the most successful and promising implementation of eHealth (e-technologies for psychiatry)?

- 1. *(What factors do you think contributes to these successes?)*

14.What are the practical challenges or problems you are facing when using eHealth in the clinical setting?

1. How do you deal with these challenges?
2. In what way might these psychiatric eHealth tools impact the doctor-patient relationships according to you?

**D. Perspectives for the future**

1. Which perspectives for a broader adoption of such technologies in standard of care?
2. Would you like/envision to have further training on integrating any of these technologies in medical care?

*(If yes: what kind of training?)*

1. Do you think doctors need to be more educated about emerging technology solutions in psychiatry?
2. What is your opinion on public health effects that could result from the implementation of any of these digital eHealth technologies?

*(Which public health problems could be solved?)*

**E. Ethical and legal challenges**

21. What special regulations or practical recommendations are needed concerning eHealth technologies use in mental health?

22. What are important ethical challenges of apps/e-Health technologies in psychiatry where regulation is needed?

23. How do you think these technologies affect the autonomy of patients with mental health disorders within the shared decision-making process?

24. What barriers do you see in implementation of eHealth technologies in psychiatric hospitals?

*(Prompting questions: may be there are economic, Is there fear of use of technology, misperception, lack of access)*

25. What approaches you’d suggest to overcome existing barriers in digital mental health care?

**F. Ending**

26. Is there anything else that I did not ask yet or that you find especially important that you would like to add? (In general or to the topic of digital tools in psychiatry?)
